# Traditional birth attendants’ experiences during the provision of post-natal care in Mopani District, Limpopo province of South Africa

**DOI:** 10.4102/hsag.v25i0.1468

**Published:** 2020-12-09

**Authors:** Roinah N. Ngunyulu, Fhumulani M. Mulaudzi, Mmampheko D. Peu

**Affiliations:** 1 Department of Nursing Science, Faculty of Health Sciences, University of Pretoria, Pretoria, South Africa; 2 Department of Nursing, Faculty of Health Sciences, University of Johannesburg, Johannesburg, South Africa

**Keywords:** Experiences, Midwife, Post-natal care, Traditional birth attendant, Rural community

## Abstract

**Background:**

South African maternity care guidelines stipulate that post-natal patients can be discharged within 6 h after delivery, provided that the condition of mothers and neonates do not require medical, surgical or obstetric attention. Hence in many instances post-natal care is rendered at home by traditional birth attendants (TBAs). Traditional birth attendants play a crucial role in the care of women during pregnancy, birth and puerperium within communities.

**Aim:**

To explore and describe the experiences of TBAs during the provision of post-natal care to mothers and their neonates in order to make recommendations to improve the quality of post-natal care delivered at home.

**Setting:**

The community hall of a selected rural traditional community was used as a setting for data collection.

**Methods:**

A qualitative, exploratory and descriptive design was used. Three focus groups were held with 26 TBAs whom were purposively selected. Data were analysed using qualitative content analysis.

**Results:**

The study confirmed two categories that included: lack of supportive working relationships between TBAs and midwives and lack of respect of TBAs, by post-natal women.

**Conclusion:**

It is evident that the TBAs experienced negative experiences. Therefore, initiation of teamwork, empowerment and confidence development are crucial to improve the working experiences of TBAs during the provision of post-natal care. Quality post-natal care might reduce maternal and neonatal morbidity and mortality rates. Teamwork between TBAs and midwives might be initiated. Continuity of care for post-natal women might be improved.

## Introduction

In South Africa, discharge of post-natal women and their neonates takes place 6 h after delivery, provided that: there is no medical, surgical or obstetric problems that require attention (National Department of Health [NDoH] 2015). In some instances, post-natal care is rendered at home by traditional birth attendants (TBAs) (Ngunyulu, Mulaudzi & Peu 2016). A TBA is ‘a person who assists the mother during childbirth and initially acquired her skills by delivering babies herself or through apprenticeship to other TBAs’ (World Health Organization [WHO] 2015).

The crucial role of TBAs is to assist women during pregnancy, labour and puerperium, which started before occurrence of modern obstetric care (Adatara et al. 2018; Aziata & Omenyo 2018; Falle et al. 2009). To date, TBAs still use common traditional practices according to their own cultural values and beliefs during the care of post-natal women and neonates at home. In Limpopo Province, South Africa, the ethnic groups are Tsonga, Venda and Northern Sotho. Traditional birth attendants promote the health and well-being of women and neonates, isolate the post-natal woman and the neonate in the room for 6 weeks to exclude evil spirits (prevent infections), exclude post-natal women from household chores to promote rest, allocate a well-known TBA to take care and prepare warm special food to maintain good nutrition and stimulate milk production for neonatal feeding, and encourage delay of sexual resumption to allow recovery of reproductive organs (Ngunyulu & Mulaudzi 2009). In Eastern Cape, dominant ethnic groups are Xhosa, Afrikaans, Sotho and Tswana. Traditional birth attendants also assist during pregnancy, delivery, post-natal care, infant feeding and family planning, using traditional and complementary health practices (Peltzer, Phaswana-Mafuya & Treger 2009). In Tshwane, Gauteng Province, ethnic groups include: Northern and Southern Sotho, Zulu, Tshwana, Ndebele, Xhosa, Tsonga, Venda, Malawi (Chichewa), Shona and Swahili Zimbabwe; they use spiritual, cultural, ancestral and family lineage to prevent and heal infants’ illness (Ramaube, Mogale & Ngunyulu 2018).

In India, TBAs place the mother and neonate together in a separate room, which promotes bonding, they wash their hands and feet before entering the room to prevent infections and prepare special warm food (Udgiri 2017). In Nepal, the rituals also include cord cutting and placenta rituals, rest and seclusion, purification, naming and weaning ceremonies, and nutrition and breastfeeding (Sharma et al. 2016). In Turkey, TBAs use practices for increasing breast milk and prevent incubus post-partum haemorrhage (Altuntug, Anik & Ege 2018).

Despite the accessibility and affordability of maternal and neonatal care services, some women still prefer assistance of TBAs during pregnancy, labour, childbirth and puerperium (Chi & Urdal 2018; Mulenga et al. 2018; Ngomane & Mulaudzi 2012). The preference and choice of maternity care by pregnant women is influenced by factors such as cultural beliefs, long distance to the nearest health facilities, disrespectful and abusive maternity care and friendliness of TBAs (Adatara et al. 2018; Fantaye, Gunawardena & Yaya 2019; Gurara et al. 2019; Mulenga et al. 2018). In Burundi and Northern Uganda, TBAs were selected as the first point of consultation by many pregnant women in times of crisis (Chi & Urdal 2018). In rural communities of Ghana, TBAs played crucial roles in home births, provision of health advice on the importance of nutrition during pregnancy and breast feeding, arrangement of transport for pregnant women to the health facilities and counselling (Adatara et al. 2018). In Nigeria, the TBAs were also involved in the provision of maternal and child health services as health promoters (Sulayman & Adaji 2019). In contrast, other women prefer hospital-based maternal healthcare services during pregnancy, labour and puerperium because of their religious beliefs (Fantaye et al. 2019).

The issue of TBAs’ involvement in maternal healthcare is not new; it was suggested in 1992 as part of WHO, United Nations Children’s Fund (UNICEF), and United Nations Population Fund’s (UNIFPA) joint statement in Geneva, Switzerland (WHO, UNIFPA & UNICEF 1992). In 2015, WHO recognised the TBAs’ important role and employed strategies to develop a collaborative relationship with TBAs to improve maternal and newborn outcomes (WHO 2015). The need for provision of quality and culturally acceptable maternal health care through involvement with TBAs was also recommended in different research studies (Adatara et al. 2018; Byrne & Morgan 2011; Gurara et al. 2019; Miller & Smith 2017; Reeve et al. 2016).

Despite recommendations from WHO and different researchers to involve TBAs in maternal health services, midwives in South Africa are not working with TBAs during post-natal care (Ngunyulu et al. 2016). Post-natal care is very crucial because during this period, women and the neonates are at risk and vulnerable to developing complications such as post-partum haemorrhage and sepsis because of infections (eds. Nolte, Marshall & Raynor 2016). The 2014–2016 report indicates that 20% of maternal deaths occur outside healthcare facilities. In addition to that, early discharge and lack of follow-up for post-natal women were identified in the report as a major and avoidable contributory factor to maternal mortality, which was overlooked in the past (NDoH 2016). It is crucial for midwives to work in collaboration with the TBAs, to identify traditional practices which might place women at risk (Sharma et al. 2016).

The available studies focused on the important role of TBAs and cultural practices, however very little is known about TBAs’ experiences when taking care of mothers and neonates. Therefore, this article reports the TBAs’ experiences during the provision of post-natal care in a selected District in Limpopo Province of South Africa.

## Research question

What are the experiences of TBAs during the provision of post-natal care to mothers and their neonates?

### Study aim

The aim of this study was to explore and describe the experiences of TBAs during the provision of post-natal care to mother and neonates to make recommendations to improve the quality of post-natal care at home.

### Research design and methods

A qualitative, exploratory and descriptive design was followed in this study (Polit & Beck 2017). This design was appropriate as it ensured in-depth knowledge and understanding of experiences of TBAs regarding the provision of post-natal care to mothers and their neonates.

### Setting

The study was conducted in a community hall at a selected rural traditional community (RTC) in Greater Giyani Local Municipality, Mopani District, Limpopo Province of South Africa. The population size of selected RTC is 14 447 and comprises of five villages (Limpopo Provincial Notice 173 of 2019).

### Study population and sampling

The population included (*n* = 26), therefore resulting in a 100% response rate of the TBAs in the community of the Mopani District. The TBAs were selected using purposive sampling. The inclusion criteria were that they should be known by the Chief as TBAs, and they should be actively involved in the care of post-natal women and neonates. All TBAs who were not known by the Chief as TBAs and were not actively involved in the care of post-natal women and neonates were excluded from the study.

As indicated in Table 1, all the participants in the study were female (*n* = 26; 100%). Their ages ranged between 30 and 80 years, with most above 50 years (*n* = 23; 88.2%), of which (*n* = 2; 7.8%) were 40–49 years and (*n* = 1; 4%) between 30 and 39 years. The majority (*n* = 20; 77%) were of the Tsonga cultural group, followed by Sotho (*n* = 4; 15%) and Venda (*n* = 2; 8%).

**TABLE 1:** Demographic variables for traditional birth attendants’.

Variables	Ranges	Number of participants (*n* = 26)	Percentages
Gender	Female	26	100
	Male	0	0
Ages	30–39 years	1	3.8
	40–49 years	2	7.6
	50–59 years	12	46
	60–69 years	8	30.7
	70–79 years	2	7.6
	80–89 years	1	3.8
Cultural background	Sotho	4	15
	Venda	2	8
	Tsonga	20	77

### Data collection

Focus group interviews were conducted by the researcher during data collection. The group dynamics of focus group assisted in expression and clarification of experiences (eds. Gray, Grove & Sutherland 2017; Moser & Korstjens 2018). The Chief of the RTC assisted with identification of TBAs and referred them the researcher. Date, time and venue of pilot testing and interviews were made with the TBAs. The purpose of pilot was to test clarity and sensitivity of research questions. The results of the pilot study were not included in the main study.

Only TBAs who agreed to participate in the study came for the focus group interview. The sitting arrangements were that of a round table, which encouraged the TBAs to be more comfortable. The title, nature and purpose of the study were explained to the TBAs. The permission to use an audiotape and to take field notes during the focus group interviews were requested and obtained from the TBAs. Informed consent forms were signed. Traditional birth attendants were made aware that they can leave the focus group anytime without any penalty against them. The data collection environment was free from noise and other interruptions.

An unstructured interview guide with one guiding question ‘What are your experiences during the provision of postnatal care?’ was used. Translation of the interview question and interviews were not carried out because the TBAs and researcher understood Tsonga clearly. Data were collected in Tsonga, which is the dominant language used in the community. Venda and Sotho speaking TBAs speak Tsonga fluently. Data collection continued until data saturation was reached during the third focus group interview. The first and second focus group interviews consisted of nine TBAs each, and the third focus group interview was conducted for data saturation confirmation and it consisted of eight TBAs. Each focus group interview lasted between 45 and 60 min.

### Data analysis

Data was analysed using three steps of qualitative content analysis (Schreier 2012). Firstly, the interviews were transcribed verbatim. Secondly, concepts and clusters were identified by careful reading and organising of data. Lastly, data were converted into smaller and more manageable units which were reviewed and retrieved. The independent co-coder who is fluent in Tsonga and English also verified categories and sub-categories. The researcher and the co-coder held a consensus meeting and agreed upon the research results. Follow-ups were made with the TBAs to verify the accuracy of agreed upon categories and sub-categories (Creswell & Creswell 2018). Then, data were displayed in their categories and sub-categories in Table 2.

**TABLE 2:** Categories and sub-categories on the experiences of traditional birth attendants’ during the provision of post-natal care.

Categories	Sub-categories
1. Lack of supportive working relationships between TBAs and midwives	1.1. Lack of post-natal care follow-up by midwives 1.2. Negative attitudes of midwives towards TBAs 1.3. Lack of confidence without supportive working relationship with midwives 1.4. Witnessing maternal deaths at home 1.4.1. TBAs doubt knowledge and skills of midwives 1.4.2. Caring for neonates after mothers’ deaths
2. Lack of respect of TBAs by post-natal women	2.1. Disrespect and undermining 2.2. Lack of mutual and trusting relationships between TBAs and the post-natal women

TBA, traditional birth attendant.

### Trustworthiness

Credibility was ensured by using focus group interviews and an unstructured interview guide, which was the most suitable data collection method and instrument, as it enabled the TBAs to freely share their experiences and learn from each other; the research question was answered and there was confidence in the collected data (Polit & Beck 2012). Pilot testing of the data collection instrument was performed, an audio recorder was used and careful examination of the transcribed data was carried out (Schreier 2012). Dependability was ensured by clearly stating the following to the TBAs: the sampling methods, inclusion and exclusion criteria, and the demographic characteristics of the TBAs, co-coding by a researcher who is fluent in Tsonga and English, and the verification, interpretation, categories and sub-categories (Creswell & Creswell 2018; eds. Gray et al. 2017; Streubert & Carpenter 2011). The confirmation of reaching data saturation is also clarified (Morse et al. 2002) as cited in (Elo et al. 2014). The collected data were accurately represented and interpreted as it is from the TBAs which ensured conformability (Polit & Beck 2012) as cited in (Elo et al. 2014).

### Ethical considerations

The permission to collect data was obtained from the following stakeholders. Ethics approval protocol number was 245/2010 and the informed consent Chief of the selected RTC and individual TBAs. Traditional birth attendants were made aware that the information provided during focus group interviews was kept confidential, and that anonymity was not possible because of a focus group interview, because they knew each other. There were no risks associated with the study; however, an onsite psychologist was hired to counsel TBAs who were emotionally and/or psychologically affected during data collection (Creswell & Creswell 2018).

## Results

Two categories and sub-categories emerged during data analysis. The categories included: lack of supportive working relationships between TBAs and midwives, and lack of respect of TBAs by post-natal women.

## Category 1: Lack of supportive working relationships between traditional birth attendants and midwives

The first category named: Lack of supportive working relationships between TBAs and midwives consisted of four sub-categories, namely: Lack of post-natal care follow-up by midwives; negative attitudes of midwives towards TBAs; lack of confidence without supportive working relationships with midwives; and witnessing maternal deaths at home.

### Sub-category 1.1: Lack of post-natal care follow-up by midwives

Supportive working relationships between TBAs and midwives is of utmost importance in ensuring transparency, sharing of knowledge and skills, which include building strength and confidence during the provision of post-natal care. The TBAs need supportive working relationships with the midwives because they are responsible for taking care of women and neonates at home after discharge from the hospitals or clinics for 6 to 8 weeks. Midwives also remain responsible and accountable for the health and well-being of the women and neonates for 6 to 8 weeks after discharge. However, the study results revealed a lack of supportive working relationships between TBAs and midwives during the provision of post-natal care. This is evident from the following quotes:

‘[*I*]t can be easy for me if I get support from the midwives, because now I am struggling with the care of the women and her neonate alone. They cannot give themselves a chance to come and see their patient at home, just to have their moral support.’ (Female, Tsonga, 39 years old)

‘[*P*]reviously I use to see a nurse riding on a bicycle, driving around the villages, visiting all the women who were discharged from the hospitals or clinics.’ (Female, Tsonga, 80 years old)

### Sub-category 1.2: Negative attitudes of midwives towards traditional birth attendants

Some TBAs expressed that midwives displayed negative attitudes towards them. Traditional birth attendants revealed that midwives do not involve them during health education of post-natal women on discharge, because they perceive that TBAs are not trained to provide maternal healthcare. Traditional birth attendants further indicated that in some instances they take post-natal women home (including high-risk) without knowledge of what happened during labour and delivery, and what is expected of them regarding continued post-natal care. This is evident in the following quotes:

‘[*I*] was shouted by a nurse, instructing me not to enter the ward because they are still giving health education to *vatswedyani* [*postnatal woman*].’ (Female, Sotho, 69 years old)

‘[*I*] did not even know where to start when I reach home with *vatswedyani* whom was reported to be very sick after delivery and was admitted a week before discharge.’ (Female, Tsonga, 55 years old)

‘[*F*]or anything I do for *vatswedyani* I remain with guilt feeling because I am aware that as traditional birth attendants we are no longer allowed to do home deliveries because the nurses regards us as no-religious, witches and people who are illiterate.’ (Female, Tsonga, 58 years old)

### Sub-category 1.3: Lack of confidence without supportive working relationships with midwives

The TBAs revealed that they did not feel confident when taking care of patients during the post-natal period. Therefore, they have feelings of inferiority, lack of confidence and they are not free to talk about the ‘indigenous’ practices they employ when caring for patients during the post-natal period. This is evident in the following quotes:

‘[*I*] am not sure whether what I am doing is right or wrong, because the nurses always shout at me for everything I do to *vatswedyani* according to my own traditional knowledge and understanding.’ (Female, Venda, 79 years old)

‘[*N*]owadays I no longer have that confidence that I use to have previously because we are being undermined by nurses, that is why we always hide everything we do for *vatswedyani* postnatal patients.’ (Female, Tsonga, 68 years old)

### Sub-category 1.4: Witnessing maternal deaths at home

In this theme, the results indicated that sometimes some TBAs had been unfortunate in seeing a woman dying on the third day after delivery from the hospital, leaving twin neonates behind. It is believed that all the patients who are discharged from the hospital or clinic after delivery are in good state of health, because they were cared for by the trained midwives. It is confirmed that some post-natal women are discharged from the hospital or clinics being in unsatisfactory conditions which lead to maternal morbidity and mortality. This is evident in the following quotes:

‘[*S*]ometimes I realise that the nurses at the hospitals and clinics do not do their work properly, because these week I came back from the hospital with my daughter-in-law who delivered twins, on arrival at home she stayed for a day, the second day she started to be weak suddenly and she fainted. I called the ambulance which came an hour later to take her back to the hospital; unfortunately, she passed away before she arrived at the hospital [*with tears dripping from her eyes*].’ (Female, Tsonga, 59 years old)

‘[*I*] saw her when she arrives home on discharge, she was not well, and because she was weak… she was not yet fit for discharge.’ (Female, Venda, 61 years old)

This sub-category consisted of two themes namely: TBAs’ doubt knowledge and skills of midwives during post-natal care and caring for the neonate after mothers’ deaths.

### Traditional birth attendants’ doubt knowledge and skills of midwives

The majority of TBAs stated that they regard midwives as highly qualified, knowledgeable and skilled professionals. They expect midwives to be able to provide quality patient care in such a way that, once they discharge a post-natal patient, they are convinced that the condition of the patient is satisfactory. However, the results confirmed that the TBAs sometimes have feelings of doubt regarding the knowledge and skills of registered midwives during post-natal care, because of the complications which occur after discharge from the hospitals or clinics. Some TBAs suggested that this type of incident can be prevented if they work as a team with the midwives during the provision of post-natal care, as this might encourage sharing of knowledge, expertise and skills. Their doubts were expressed as follows:

‘[*I*] think they left some products of conception inside the uterus, they were expected to compress the abdomen until all the products are expelled, because they are dangerous to the life of a woman as they cause infection.’ (Female, Tsonga, 74 years old)

‘[*I*]f the midwives were willing to work with us, we were going to show them how we examine *vatswedyani* and remove all the products of conception in a traditional way so that there is no infection, but they do not even want to hear anything from a TBAs, because they think they know everything.’ (Female, Tsonga, 57 years old)

### Caring for the newborn babies after the mother’s death

The TBAs confirmed that there were women who died during the post-natal period after discharge from the hospitals or clinics, leaving the newborn infants to be cared for care by them. They further indicated that this was a serious challenge because they struggled alone, without support visits from midwives. As elderly people, they no longer had the physical strength to provide necessary care for the newborn infants. This is evidenced in the following quotes:

‘[*N*]ow I am faced with the responsibility of taking care of twin infants, because the mother passed away on the second day after discharge from the hospital [*continued crying*].’ (Female, Tsonga, 69 years old)

‘[*I*]’m struggling to raise a new-born baby whose mother passed away two weeks after delivery… his father is also in a critical condition at the hospital.’ (Female, Sotho, 46 years old)

## Category 2: Lack of respect of traditional birth attendants by post-natal women

Category 2 consisted of two sub-categories, namely: ‘disrespect and undermining’, ‘Lack of mutual and trusting relationships between TBAs and post-natal women’.

### Category 2.1: Disrespect and undermining

Culturally, a woman at childbearing age is expected to show respect to elderly people including the TBA who is taking care of her during the post-natal period. Post-natal women are culturally obliged to follow the instructions and advice given by the TBA as a mark of respect. However, the TBAs expressed concerns regarding the treatment they received from some of the post-natal women, because they are no longer showing respect. The TBAs further revealed that they feel disrespected and undermined because the post-natal women do not agree to follow their advice during the post-natal period. The TBAs had previously been expected to make a final decision regarding the care of post-natal woman and the newborn, but now it is no longer the case. This is evident in the following quotes:

‘[*P*]reviously I use to keep the woman and the new-born baby in my hut until the end of the second month, but now things have changed. When the woman and the baby are discharged from the hospital or clinic, the father is the one who is carrying the baby home, so I just keep quiet because even if I talk, they do not listen to me.’ (Female, Sotho, 53 years old)

‘[*T*]he way of doing things differ from one family to another, with me in my family. On coming back from hospital with the woman after delivery I don’t do anything because I am aware that they regard me as a witch, so I am afraid that if I keep this woman in my hut and something happen to the baby or mother, they will conclude that I bewitched them, so I just keep quiet because I do not want to be killed by their husbands.’ (Female, Tsonga, 58 years old)

‘[*W*]hen I request her to come to my hut with the new-born for isolation against evil spirits, she said that: “Clinic sisters told me not to take any other advice except the advice given at the clinic or hospital’’.’ (Female, Tsonga, 61 years old)

‘[*Y*]oung men are dying every day because they do not follow, he taboos during postnatal period … delayed resumption of sexual relations.’ (Female, Tsonga, 57 years old)

### Sub-category 2.2: Lack of mutual and trusting relationships between traditional birth attendants and post-natal women

Some TBAs further revealed that they feel that they are no longer trusted by the post-natal women. Post-natal women do not allow TBAs to hold the neonates, which makes them feel guilty, because the post-natal women think that they might poison the neonates, which impact their relationship. This was evident in the following quotes:

‘[*S*]he does not even allow me to come closer or to hold the new-born baby, she keeps the baby away from me.’ (Female, Sotho, 48 years old)

‘[*W*]hen I ask her the reason for not allowing me to hold the baby, she told me that, she is afraid that I will put black stuff on the baby’s head and the sisters at the hospital said that it is dangerous for the baby.’ (Female, Tsonga, 69 years old)

## Discussion

The TBAs confirmed that they experience lack of supportive working relationships with midwives, which is a serious concern because both practitioners are directly involved with care of post-natal patients for 6 to 8 weeks after delivery. In case the post-natal woman complicates at home within 6 to 8 weeks of discharge, it remains the responsibility of the midwife to account for the health and well-being of the woman and the neonate. Midwives are obliged to facilitate continuity of care through follow-up and support of women, neonates and family during pregnancy, labour and the post-natal period (SANC 2013). Therefore, midwives are responsible for their attitudinal changes, in order to initiate therapeutic working relationships with the TBAs, which was recommend by many researchers worldwide (Adatara et al. 2018; Amutah et al. 2017; Byrne & Morgan 2011; Gurara et al. 2019; Miller & Smith 2017; Reeve et al. 2016; WHO 2015). As a result, active involvement of TBAs might be ensured because midwives will be able to advise TBAs about danger signs during the post-natal period that need urgent referral. Therefore TBAs might feel supported and this will yield a positive working experience, improved confidence, resulting in provision of quality care and improved post-natal care outcomes.

Traditional birth attendants also confirmed that they experience negative attitudes from midwives, who exclude them from health education sessions of post-natal women after discharge because they regard them as being illiterate. Based on the results of this study midwives at the clinic and hospital close to this RTC do not appear to value the role of TBAs during the provision of post-natal care, hence they are reluctant to work in collaboration with TBAs. The TBAs are one of the categories of the traditional health practitioners which must undergo education and training at any accredited training institution, as outlined in the Government Gazette No 39358 of 03 November 2015 as amended (NDoH 2015). However, the process towards the training is too slow. As a result, the TBAs in South Africa are not trained, hence they lack confidence, therefore their practices are undermined and regarded as unsafe by the midwives (Ngunyulu et al. 2016). In Ethiopia, there is a lack of respectful midwifery care, formal and trusting working relationships between midwives and TBAs. As a result, the majority of pregnant women choose to deliver at home. This is evidenced by 70% of deliveries occurring at home conducted by untrained TBAs (Gurara et al. 2019). In addition to that, in Zambia, home deliveries by TBAs continued despite that the Zambian government discontinued the training of TBAs and banned homebirth by TBAs, as they were regarded as the major contributory factor to maternal mortality (Cheelo, Nzala & Zulu 2016). In contrast, Kayombo (2013) indicated that training of TBAs might have a positive impact on the promotion of reproductive health and reduction of maternal morbidity and mortality in Sub-Saharan Africa.

Regarding the care of orphaned neonates by TBAs in association with maternal deaths which some are witnessed at home. This was confirmed in the saving mothers report (2014–2016), that more than 36% of deaths occurred in the post-natal period, and most of the women who died were discharged after delivery with abnormal vital signs. Furthermore, 57.0% of avoidable maternal deaths occurred because of delay in accessing medical help as a result of unavailability of transport from home to the facility (NDoH 2018). This is applicable to the selected RTC because four out of five villages within the RTC do not have a local clinic and it is more than 10 kilometres away from the nearest clinic or hospital, hence they end up witnessing maternal deaths at home because of delays in reaching health facilities in case of an emergency. The neonates of all women who died as indicated in the reports are left under the care of TBAs, family members or relatives. The issue of doubting of knowledge and skills of midwives by TBAs was also confirmed by the saving mothers reports of 2014–2016, which pointed out that 25% of all maternal deaths with avoidable factors are associated with lack of knowledge and skills of nurses (NDoH 2014–2016).

Traditional birth attendants confirmed that they also experienced lack of respect, being undermined and resulted in lack of mutual and trusting relationships between TBAs and post-natal women. This was evidenced by a refusal of health advice by post-natal women and not allowing TBAs to hold their neonates. In contrast, some women prefer the assistance of TBAs during pregnancy, labour, childbirth and puerperium, because they experienced abusive and disrespectful care by midwives as opposed to friendly care by TBAs (Chi & Urdal 2018; Mulenga et al. 2018; Ngomane & Mulaudzi 2012). In Pakistan, the TBAs could assist midwives with normal deliveries and refer high-risk pregnant women to the hospital (Shaikh et al. 2014). Meanwhilst the TBAs in Northern Uganda were no longer permitted to conduct deliveries post conflict (Chi & Urdal 2018). Despite the vital role that the TBAs played in the community with positive outcomes, some health governments including South Africa still cannot trust their TBAs’ practices.

## Strength and limitations

The strength was that the research design and the method of the study were clearly explained which enables other researchers to replicate it in other contexts. The study was only conducted in the Mopani District, Limpopo Province of South Africa; therefore the results could be used as a guide for other communities.

## Recommendations

### Midwifery practice

- Public policy makers should initiate a collaborative working relationship with the TBAs, to share knowledge and skills, learn from each other and empower each other.
- Support the TBAs by involving them in a health education session of post-natal patients on discharge, to promote mutual and trusting relationships between the TBAs and post-natal patients; this will prevent conflicts of undermining each other at home.
- Ensure continuity of care, by giving a formal report about the condition of the patient during pregnancy, labour and delivery to the TBAs, and post-partum danger signs, that need urgent medical assistance, to improve the quality of post-natal care and prevent morbidity and mortality rates.
- The primary health care sector should strengthen their relationship with TBAs and community health workers.

### Training

- The midwifery curriculum should include traditional practices, values and beliefs, to empower midwives with cultural knowledge, awareness, sensitive, competence and compassion.
- The NDoH should facilitate training of TBAs to empower them with knowledge and skills that will improve their confidence and instil positive working experiences.

## Research

- More nursing research should be conducted to produce strategies to integrate TBAs into the midwifery healthcare system.

## Conclusion

It is evident that the TBAs had negative experiences only during the provision of post-natal care at home. The negative experiences include lack of supportive working relationships with midwives and lack of respect of TBAs by post-natal women. Teamwork between the midwives and TBAs is crucial in ensuring continuity and quality of post-natal care to prevent avoidable contributory factors to maternal mortality and morbidity.
